# Quarantine, isolation and the duty of easy rescue in public health

**DOI:** 10.1111/dewb.12165

**Published:** 2017-09-18

**Authors:** Alberto Giubilini, Thomas Douglas, Hannah Maslen, Julian Savulescu

**Keywords:** easy rescue, isolation, public health, quarantine

## Abstract

We address the issue of whether, why and under what conditions, quarantine and isolation are morally justified, with a particular focus on measures implemented in the developing world. We argue that the benefits of quarantine and isolation justify some level of coercion or compulsion by the state, but that the state should be able to provide the strongest justification possible for implementing such measures. While a constrained form of consequentialism might provide a justification for such public health interventions, we argue that a stronger justification is provided by a principle of State Enforced Easy Rescue: a state may permissibly compel individuals to engage in activities that entail a small cost to them but a large benefit to others, because individuals have a moral duty of easy rescue to engage in those activities. The principle of State Enforced Easy Rescue gives rise to an Obligation Enforcement Requirement: the state should create the conditions such that submitting to coercive or compulsive measures becomes a fundamental moral duty of individuals, i.e. a duty of easy rescue. When the state can create such conditions, it has the strongest justification possible for implementing coercive or compulsive measures, because individuals have a moral duty to temporarily relinquish the rights that such measures would infringe. Our argument has significant implications for how public health emergencies in the developing world should be tackled. Where isolation and quarantine measures are necessary, states or the international community have a moral obligation to provide certain benefits to those quarantined or isolated.

## 1 INTRODUCTION

Quarantine and isolation are sometimes used to contain or minimise infectious disease outbreaks, particularly in the developing world. For instance, because at the time of the 2014‐2015 Ebola outbreak no available vaccine had been tested on humans, isolation and quarantine were widely used to reduce Ebola transmission in West Africa.^1^ One example is provided by the case of the village of Sella Kafta in Sierra Leone, where the entire village of 1000 inhabitants was placed under quarantine for 3 weeks after one woman died of Ebola. On that occasion, quarantine measures included a curfew in which people were not allowed to move from one house to the other, which was enforced by soldiers and the police.^2^

Such measures can be morally controversial. In this paper we consider when, why and under what conditions the state has the strongest justification possible for implementing quarantine and isolation.

We will take it that quarantine and isolation can be justified, and indeed morally mandatory, when the expected benefit to others and to society, in terms of infectious disease prevention or limitation, outweighs the expected costs, including the moral costs of coercion and compulsion, and satisfies three further constraints. However, we will argue that authorities ought to implement quarantine and coercion in such a manner that they have the *strongest* justification possible for those measures. Further, we take it that the justification for these measures is, other things being equal, stronger when quarantined or isolated individuals have a *moral duty* to submit to those measures. We argue that individuals fall under a duty of easy rescue, i.e. a moral obligation to benefit others, or to prevent harm to others, when doing so entails a small cost to them. We distinguish two types of easy rescue that have been presented in the philosophical literature, namely a comparative and an absolute type; we argue that individuals have an uncontroversial duty of easy rescue of a third type, and that, *in certain circumstances*, such duty implies that individuals have a duty to submit to quarantine or to isolation. Thus, the state can in some cases fulfil its requirement to act with the strongest justification possible by ensuring that the cost individuals bear for being quarantined or isolated is small, so that their submitting to quarantine or isolation fulfils the conditions of an easy rescue. Finally, we outline how, in concrete terms, this could be achieved, with particular reference to the ethical obligations of local authorities and of the international community in the case of quarantine and isolation measures implemented in poor countries.

Before presenting the argument in more detail, it will be useful to say something more about nature of and moral issues presented by quarantine and isolation.

## 2 QUARANTINE AND ISOLATION AS COERCIVE OR COMPULSORY MEASURES

Quarantine and isolation are two measures that can be used to prevent or minimize the impact of infectious disease outbreaks

‘by preventing exposure to people who have or may have a contagious disease. Isolation separates sick people with a contagious disease from people who are not sick. Quarantine separates and restricts the movement of people who were exposed to a contagious disease to see if they become sick’^3^

Quarantine is typically in two respects more ethically problematic than isolation. First, it involves the confinement of individuals who might not be infected. For example, in the case mentioned in the introduction, an entire village in Sierra Leone was quarantined because any individual *might* have been exposed to Ebola. Secondly, it typically forces people who have not been infected to be in spatial proximity to those who have been infected, thereby increasing their chances of becoming infected.

Quarantine might be mandated for people who have been exposed to a disease and refuse compulsory medical treatment,^4^ as well as when such treatment is not available,^5^ as in the case of the 2014‐15 Ebola outbreak in West Africa. Often, isolation and quarantine involve not only physical confinement, but also cognitive, affective and spiritual isolation due to the limitations in the interactions with, respectively, health workers, relatives, and religious leaders.^6^

The restrictions on freedom involved in quarantine and isolation are sometimes described as instances of coercion.^7^ In philosophical discussion, coercion is often taken to involve an actor – in this case the state or some health authority – *forcing* a person to do as the actor wants.^8^ “Forcing” someone might however mean two different things, i.e. it might mean making alternative options extremely unappealing, or it might mean removing these options entirely—that is, rendering them impossible. According to an influential view propounded by Joel Feinberg,^9^ and which we accept, only the former constitutes coercion properly understood; when alternatives are rendered *impossible* we should instead speak of compulsion. Compulsion occurs when, as in the case mentioned in the introduction, soldiers or the police are deployed to guarantee citizens’ compliance. In public health, compulsion is often reserved for cases where public health issues are seen as a security threat for a country.^10^

Quarantine and isolation plausibly involve coercion or compulsion, and coercion and compulsion are plausibly *pro tanto* wrong—that is, wrong absent defeating considerations. Thus, it is normally thought that these interventions can only be justifiably imposed if there is a strong case in favour of them. But in many instances in which quarantine and isolation are adopted, and particularly when they are adopted as emergency measures, there *is* a strong case in favour. These interventions are normally deployed to prevent potentially devastating consequences of infectious disease. Such consequences are discussed in the next section.

## 3 BENEFITS OF QUARANTINE AND ISOLATION

One reason in favour of quarantine and isolation is that they *can* be very effective in protecting or restoring public health. For example, the experience of the 2003 SARS outbreak demonstrated that infectious diseases like SARS can sometimes be contained if a series of timely measures are implemented, including the early identification of infected people and contact tracing as well as timely quarantine and isolation measures.^11^ Mathematical models have suggested that some emerging infectious diseases at the initial stages can be successfully contained when there is a high probability that an asymptomatic but contagious individual will be placed in quarantine before developing symptoms.^12^ Granted, the benefits of quarantine and isolation depend on the context and the their effectiveness in particular cases has often been contested.^13^ In what follows we will be assuming, supported by the evidence and data provided, that quarantine and isolation can at least in certain cases be effective; we intend our arguments to apply only to such cases.

To the extent that quarantine and isolation are effective in protecting or restoring public health, they can also contribute to protecting human and national security. Human security is to be understood as “safety from constant threats of hunger, disease, crime and repression”^14^ . It includes core aspects of a good human life such as health, safety, employment and freedom from fear.^15^ These aspects are all affected by the quality of public health, and as such might depend on effective and timely implementation of coercive or compulsory public health measures. The same can be said for national security, which includes safety from military threats to states, but also ‘non‐military issues, including the economy and trade, the environment and infectious diseases’.^16^ For example, as the prevalence of infectious diseases increases, indicators of state capacity such as gross national product per capita, government expenditures, military spending per capita and enrolling rate in secondary school decrease.^17^

Indeed, infectious diseases can also have a considerable financial cost, which needs to be factored in when weighing pros and cons of timely and effective implementation of public health measures. Consider again the Ebola virus outbreak of 2014 and 2015. The World Bank estimated that Guinea, Liberia and Sierra Leone lost ‘at least US$ 2.2 billion in forgone economic growth in 2015 as a result of the epidemic’.^18^ The same is true for the 2003 SARS outbreak: the total cost of the SARS epidemic for the world economy has been estimated to be at least US$40 billion.^19^

It is important to point out that the abovementioned health, security and economic benefits of quarantine and isolation can generate reasons in favour of quarantine or isolation even if there is uncertainty regarding the effectiveness of such measures in a given context. When making decisions in conditions of uncertainty, we need to assess the *expected* benefits and harms of a certain choice (say, implementing quarantine measures) against the *expected* benefits and harms of a different choice (say, not implementing quarantine measures). The expected benefits or expected harms are the products of the magnitude of, respectively, the benefit or harm in question and of the probability of their occurrence.

The expected health, security and economic benefits of quarantine and vaccination need to be weighed against any expected health, security and economic costs, as well as any ‘moral’ costs intrinsic to coercion and compulsion. It is plausible that, in some cases, the expected benefits will outweigh the expected costs in the sense that the state has stronger reasons to pursue the expected benefits than to avoid the expected costs. In such cases, we believe the state is justified in implementing quarantine or isolation—that is, it is at least *morally permissible* for the state to do so.

Moreover, because states have a duty to protect public health as well as national and human security, they have an at least prima facie *duty* to implement coercive and compulsory measures when these are necessary to protect public health and human and national security. The central question of this paper is whether the simple appeal to such a duty of the state represents the strongest justification possible for implementing coercive and compulsory measures. Our answer will be that it does not, and that this duty of the state needs a further argument in its support in order to yield the strongest justification possible for coercive and compulsory state interventions in public health. Before explaining why the simple appeal to the duty of the state does not provide the strongest justification possible, and what type of consideration needs to be added in order to have such a justification, it will be useful to introduce, in the next section, one ethical theory that can provide a moral justification for the duty to implement coercion and compulsion in public health, namely a constrained form of consequentialism.

## 4 THE CONSEQUENTIALIST JUSTIFICATION FOR COERCION AND COMPULSION IN INFECTIOUS DISEASE CONTROL

Consequentialism, on one standard formulation, is the view that an action is morally permissible if and only if it has consequences at least as good as any alternative action, and morally mandatory if its consequences are better than the consequences of any alternative action. A common variant of consequentialism, adapted to conditions of uncertainty, holds that the goodness of the set of consequences associated with an action is to be understood in terms of expected value. Suppose an agent faces a choice between a range of possible actions, each of which could produce a range of possible outcomes. The expected value of a given outcome is given by the probability of it occurring if the agent performs the action in question multiplied by the value of the outcome. The expected value of a given action is the sum of the expected values of the outcomes that it might produce. According to this variant of consequentialism, an action is right if and only if its expected value is at least as great as that of any alternative action.^20^

Thus, this form of consequentialism implies that as long as the expected positive value of a public health benefits (and any knock‐on social or economic benefits) brought about by coercive or compulsory public health measures is no less than the expected negative value of temporary right infringements (and any knock‐on social, economic or medical costs), such public health measures are morally permissible, even when they entail some degree of coercion or compulsion; and when the net expected positive value of coercive and compulsory public health measures is superior to the net expected positive value of alternative measures, coercive and compulsory public health measures are morally obligatory.

However, consequentialism, as characterised above, also has some intuitively unpalatable implications. For example, it is intuitively not justifiable to isolate or quarantine people who have contracted or have been exposed to viral gastroenteritis, even if this is expected to have net beneficial consequences and to be the most cost‐effective measure of preventing contagion. This principle may thus need to be constrained in various ways, and indeed a range of possible constraints have been proposed. We call the resulting version “constrained consequentialism”. For example, requirements of proportionality are typically appealed to in order to limit the application of simple versions of consequentialism.^21^ Similar constraints are also commonly invoked by ethical guidelines regulating public health measures.^22^ In what follows, we will make what seem to us to be reasonable and intuitive assumptions regarding the constraints to which consequentialism should be subject to when applied to assess the morality of coercive or compulsory measures: we will assume that, in addition to bringing about more positive than negative value, morally permissible forms of coercion and compulsion in public health must satisfy three constraints, namely:

1. The *severity* of the harm to be prevented or contained through coercive or compulsory public health measures should be significant. For example, it does not seem justifiable to quarantine individuals who might have been exposed to viral gastroenteritis, because the harm of gastroenteritis is not severe enough, but it might have been justifiable to quarantine the village of Sella Kafta in Sierra Leone in order to detect symptomatic and therefore contagious cases of Ebola at the onset.
2. Less restrictive measures for preventing or containing the infectious disease should be preferred to more restrictive measures; for example, ‘all other things being equal, a policy that provides incentives for persons with tuberculosis to complete their treatment until cured will have priority over a policy that forcibly detains such persons in order to ensure the completion of treatment’.^23^
3. There should be *proportionality* between the public health measure implemented and the threat to public health. In general terms, the more harmful a disease is, the more restrictive the measures that a state may be justified in imposing are. For example, it seems plausible to say that isolation is not a proportionate measure against viral gastroenteritis, although it would be effective, while it seems proportionate against Ebola or SARS: the significant threat that such infectious diseases pose justifies imposing a certain level of coercion or compulsion in order to avoid or contain outbreaks.

We will take it that, on the basis of constrained consequentialism, it can be morally permissible for a state to impose coercive and compulsory public health measures that satisfy these constraints and are expected to produce as much net value as any alternative that also satisfies the constraints; and that it is morally mandatory for a state to impose coercive and compulsory public health measures that satisfy these constraints and that are expected to produce more net value than alternatives. Thus, on some occasions, considering the significantly bad consequences of certain infectious diseases that can be prevented through effective quarantine and isolation measures, a state duty to impose quarantine and isolation would be supported by constrained consequentialism.

## 5 THE DUTY OF EASY RESCUE IN PUBLIC HEALTH

But what about the moral obligations of each single individual to contribute to protecting public health? The question is relevant in discussing the justification for coercive or compulsory state interventions for the following reason. We can assume that the state should have the strongest justification possible for implementing restraining measures that temporarily infringe upon certain individual rights. But it is doubtful that the (constrained) consequentialist considerations we have provided so far, by themselves, represent the *strongest* justification possible for restricting freedom of movement and association, although they may be strong enough to support a duty to implement quarantine and isolation in certain circumstances. To see this, notice that, in cases where our above constrained consequentialist justification applies, the individuals who are coerced or compelled may have no moral duty to submit to such measures.

Now, it is commonly and in our view plausibly thought that a state has a stronger basis for coercing or compelling its citizens when its citizens have an independent moral obligation to do that which the state is compelling them to do than when it does not.^24^ In the former cases, the state is merely coercing or compelling individuals to do what they in any case ought to do. On this view, the state's justification for implementing quarantine or isolation would be stronger if individuals had a *moral duty* to submit to these measures. But do they have such a duty? For example, did the inhabitants of the village of Sella Kafta in Sierra Leone have a moral duty to submit to quarantine? And therefore, was the justification for imposing the quarantine the strongest possible? In this section we argue that, *in certain circumstances*, there is a duty to submit to quarantine or isolation.

The proponents of a wide range of moral theories could accept that there is a duty of easy rescue, according to which, if we can do something that would entail some significant benefit to others at a small cost to us, we have a moral obligation to do it. Peter Singer famously illustrated a version of the duty of easy rescue through his example of the drowning child: if I walk past a pond and notice a child drowning in it, I have a moral obligation to save the child even if this means getting my clothes muddy.^25^

In the same way, considering the grave harms that even a single individual can cause through spreading an infectious disease, and that can in some cases be prevented through quarantine and isolation, enduring the constraints involved in quarantine and isolation might, *depending on the circumstances*, represent a moral obligation that is justified by a duty of easy rescue. The relevant question is if and when the cost to individuals is small enough. For example, a small cost might be temporarily forgoing international air travel.

However, quarantine and isolation can be far more burdensome than forgoing international travel. In Singer's example of the drowning child, the cost to the rescuer is small: the individual does not suffer significantly from getting her clothes mudded. By contrast, in some instances of quarantine and isolation, the personal cost can be great even when its moral relevance remains small *compared* to the moral significance of the harm prevented. For example, Ebola‐exposed families who were subject to quarantine measures in West Africa during the 2014‐15 outbreak suffered significantly from stigmatization and loss of livelihoods,^26^ as well as possible increased exposure. A survey conducted by the WHO and the Liberia's Ministry of Health found that during the outbreak many families in quarantine did not receive food supplies and that communication between quarantined people and their families was often impossible.^27^ In some cases even access to water and sanitation facilities could not be guaranteed for people in quarantine.^28^ These conditions do not seem compatible with quarantine or isolation being forms of “easy rescue”. However, as we have seen in the previous sections, the great harm that can be prevented through quarantine and isolation certainly is of great moral importance, and therefore the moral relevance of the infringement of individual rights might still be small *when compared to that*.

Some formulations of the duty of easy rescue do require that individuals be subject to quarantine and isolation in such cases. One such formulation has been provided by Singer, according to whom the example of the drowning child suggests that ‘if it is in our power to prevent something bad from happening, without thereby sacrificing anything of *comparable* moral importance, we ought, morally, to do it’.^29^ Call this the duty of comparatively easy rescue. According to it, there is a moral obligation to sacrifice something when the moral relevance of this sacrifice is small *compared* to the moral relevance of the good to be achieved, rather than when the cost is small in absolute terms. So that principle can still morally demand making moderate or even great sacrifices, such as submitting to quarantine or isolation even when the conditions are very harmful to individuals, where there is something of significantly greater moral importance at stake. One might think that the duty of comparatively easy rescue is too demanding. The state might well have reasons to enforce coercive and compulsory public health measures, but it is more difficult to accept the idea that individuals have a moral obligation, based on a duty of *easy* rescue, to submit to quarantine and isolation, given the great cost involved. If we think individuals have uncontroversial moral duties of easy rescue, the general formulation of the duty of easy rescue needs to be less demanding than the one offered by Singer.

A less demanding and easier to accept formulation of the duty of easy rescue is one that takes into account also the costs to individuals in *absolute* terms, and not only the relative size of the cost compared to the benefit that could be gained. A milder duty of this sort would morally demand benefitting others or preventing harm to others only when the individual cost is small enough. One such formulation has been suggested by Tim Scanlon, according to whom ‘[if] you can prevent something very bad from happening (…) by making only a slight (or even moderate) sacrifice, then it would be wrong not to do so’.^30^ Call this the duty of absolutely easy rescue.

We favour a third formulation of the duty of easy rescue that combines both comparative and absolute elements, as follows:

If the cost (including foreseeable risk of significant disability or death) to someone of performing an action X (or of refraining from performing an action Y) is sufficiently small to be reasonably bearable, and the resulting benefit to other people (or harm that is prevented) is large relative to the cost, then the agent ought to do X (or not do Y).

This formulation of the duty retains the idea that there should be proportionality between the individual cost and risk on one side and the benefit to other people on the other, such that the greater the cost, the greater the benefit required for the agent to fall under a duty; however, the formulation also places an absolute upper limit on the cost that must be borne by the agent: the cost should be reasonably bearable (we understand ‘reasonably bearable’ such that whether a cost is reasonably bearable is independent of the size of the benefit that will be brought about by bearing it). In combining both absolute and comparative constraints, this formulation generates a narrower duty than either of the previous formulations, and should thus be more broadly accepted. It is for this reason that we adopt it.

Now, the relevant question is: would the duty of easy rescue, thus formulated, impose a moral obligation on individuals to submit to quarantine or isolation? The answer will depend on the circumstances.

It is plausible to assume that loss of livelihoods, stigmatization, and lack of food, water and sanitation—circumstances that, as noted above, often occurred in the case of quarantine measures during the Ebola outbreak in West Africa—do not constitute forms of “easy rescue”, especially when they are endured for a significant period of time. That being so, it seems that the duty of easy rescue, as we have interpreted it, does not impose a moral obligation on individuals to submit to quarantine and isolation in those circumstances. Therefore, *in such circumstances*, the general justification for coercing or compelling people to submit to quarantine or isolation is weaker than it would have been if individuals had a moral duty to submit to quarantine and isolation. However, even if that is the case, such circumstances are a contingent, and not a necessary feature of quarantine and isolation. If the conditions of those in quarantine and isolation were improved—for example, if individuals were provided with food, water, sanitation and medical assistance (in order to reduce the risk of contagion), psychological counselling, adequate financial compensation for any loss of livelihoods—submitting to quarantine or isolation might represent a form of rescue that is easy in both comparative and absolute terms. Therefore, our formulation of the duty of easy rescue would imply that individuals are under a moral duty to submit to such measures.

In section 6, we explain how our argument to this point is consistent with a principle that one of us has defended elsewhere, i.e. with what we here call the principle of “state enforced easy rescue”. As we shall see, the principle of state enforced easy rescue picks out the cases in which, according to our argument above, the state has a particularly strong justification for imposing quarantine or isolation.

In section 7, we will examine how states may comply with the requirement we have defended above: that states impose quarantine or isolation in such a way that they have the strongest justification for doing so. We also argue that, when states cannot fulfil this requirement, as might happen in the case of developing countries, it is the international community that has the corresponding obligation to provide the assistance that is necessary to meet the duty enforcement requirement.

## 6 THE PRINCIPLE OF STATE ENFORCED EASY RESCUE

In the previous sections, we introduced the idea that states should impose quarantine and isolation in such a manner that they have the strongest justification for doing so. We also introduced the individual duty of easy rescue, and argued that, at least in certain circumstances, submitting to quarantine or isolation might fall within the scope of this duty.

When the duty of easy rescue generates a duty to submit to quarantine and isolation, these measures can be conceptualized as enforcements of a fundamental individual moral duty, giving the state a stronger justification for implementing them than they would have in the absence of such an individual duty—in many cases, this may be the strongest justification available.

Our argument to this point is consistent with a general principle governing public policy that we call the principle of state enforced easy rescue, and which has been formulated by Julian Savulescu as follows:

‘[w]hen the cost to us of engaging in some activity is small (..), and the harm to others which is prevented is great, the state may permissibly compel us to engage in that activity’.^31^

We intend the “us” included in this principle to refer to any individual, rather than to individuals considered in aggregation; therefore, the small cost mentioned in the principle is the cost to any individual, rather than the aggregate cost of a certain activity. We also interpret the requirement that the cost be ‘small’ as entailing that the cost be small enough in absolute terms to be reasonably bearable, and that it is small relate relative to the harm averted.

Thus interpreted, this principle represents the strongest justification possible for implementing quarantine and isolation measures. The principle implies that the state may permissibly compel individuals to do something if the benefit to other people, including the harm that is prevented, is significant and if the cost to any individual is comparatively and absolutely small. Since in such cases individuals would, according to the individual duty of easy rescue that we introduced above, have a moral duty to do what the state compels them to do, the principle of state enforced easy rescue picks out cases in which there is an especially strong justification for coercion or compulsion.

But how can the state ensure that the implementation of quarantine or isolation satisfies the principle of state enforced easy rescue? As we will see in the next section, the state can—and should, according to the “duty enforcement requirement” we will introduce—take steps to create the conditions such that the costs to individuals of quarantine and isolation are small enough, where they might otherwise not have been.

## 7 THE DUTY ENFORCEMENT REQUIREMENT AND THE DUTIES OF THE STATE

As we have said above, it is plausible that a state should have the strongest justification possible for subjecting individuals to coercive or compulsory measures such as quarantine and isolation. The justification for quarantine and isolation is, other things being equal, stronger when state intervention takes the form of a state enforced easy rescue, i.e. when the individuals subject to quarantine and isolation have a duty to submit to them, for example because doing so falls within the scope of a duty of easy rescue. Thus, we can say that, if possible, the state ought, other things being equal, to make it the case that its citizens have a moral duty, such as the one grounded in a duty of easy rescue, to submit to quarantine and isolation. We call this the duty enforcement requirement.

One way that the state could comply with the duty enforcement requirement is to provide people in quarantine and isolation with what they need in order to ensure that submitting to quarantine and isolation is a form of easy rescue. It may be able to do this, for example, by providing people in quarantine or isolation with food, access to clean water and sanitation (which, as we have seen, have not always been guaranteed by local governments in certain developing countries), psychological counselling, means to easily communicate with loved ones, protection from cross infection, prompt medical treatment if the condition develops and anything that would make the cost and the risk to them small enough to be reasonably bearable.

Thus, the duty enforcement requirement provides an independent ethical justification for a principle of reciprocity in public health, which specifies what a state owes to individuals in exchange for their submitting to restraining measures for the sake of the public good.^32^ As put by Diego Silva and Maxwell Smith, ‘reciprocity maintains that when an individual is subject to a limitation on their human rights or freedoms for the sake of a public health emergency, the State must support and compensate that individual for his or her loss, so they are not unduly harmed’;^33^ according to them, the principle of reciprocity demands, for example, that ‘society provides resources such as food and water to those burdened by restrictive measures like isolation or quarantine’.^34^ We think the duty enforcement requirement has similar implications, and that it can justify a principle of reciprocity in public health.

Granted, often states would not be able to provide the assistance that is owed to constrained individuals. Developing countries in particular would struggle to fulfil this obligation. In such cases, we claim that it is the international community that should provide the necessary support to the state in question. The responsibility to make it the case that the cost to people in quarantine or isolation is sufficiently small is a responsibility that falls on all of us, regardless of where an infectious disease outbreak occurs or is likely to occur, Thus, for example, Western societies have a responsibility to contribute to making public health measures in the developing world easy to bear for those affected, by funding the necessary psychological, material and social support that constrained individuals need.

In the case of the quarantine of the village of Sella Kafta, for instance, the WHO reports that ‘[d]ifferent organizations provided food, water supplies, social support, educational support for children, even solar powered telephones and assistance with farms so that crops were not left to rot during the growing season’.^35^ These represent good examples of what local governments and the international community ought to do in order to support individuals in quarantine or isolation in developing countries, so as to ensure that submitting to quarantine or isolation constitute forms of easy rescue.

## 8 CONCLUSIONS

The aim of this paper has been to answer the questions as to when, why and under what conditions the state has the strongest justification possible for implementing quarantine and isolation as means of containing or preventing infectious diseases.

As for *when*, we have argued that when infectious diseases threaten public health, national and human security, or the economy of entire countries, as was the case with the 2014‐15 Ebola outbreak in West Africa, quarantine or isolation may be—*all things considered—*justified by (constrained) consequentialist considerations if it can be expected that they would be effective in preventing or containing contagion, but that the justification is the strongest possible when states can guarantee that certain individual basic needs of those in quarantine or isolation are met.

As for *why* quarantine and isolation are justified, we have argued that the justification for state interventions is stronger than the one offered by our above‐described version of constrained consequentialism if individuals have a moral obligation, based on a duty of easy rescue, to submit to such measures. This involves a refinement of our above‐described variant of constrained consequentialism in that it adds a further constraint: where it can do so, the state must fulfil the duty enforcement requirement. It can sometimes do so by ensuring that the quarantine or isolation measures impose only costs that are both comparatively and absolutely small, such that individuals have a duty to submit to them.

And so, finally, as for *the conditions* under which the justification for quarantine and isolation is stronger, we have argued that a state has the strongest justification possible for quarantine and isolation if, where it can do so, it fulfils its duty to make the burden of such measures easy enough to bear for those affected, or, in case of developing countries that would struggle to meet this requirement, if the international community provides the means that are necessary for the state to fulfil this obligation. In this way, the state is in the position to present its intervention as the enforcement of an individual moral duty of easy rescue, thus fulfilling the duty enforcement requirement.

## CONFLICT OF INTEREST

No conflicts declared.

## Notes

1 Kucharski AJ, et al. Measuring the impact of Ebola control measures in Sierra Leone. *Proceedings of the National Academic of Sciences of the United States of America* 2015; 112 (46): 14366‐71.

2 See, for example, BBC, ‘Sierra Leone village in quarantine after Ebola death’, 4 Sept 2015, Available at http://www.bbc.co.uk/news/world-africa-34151494 [Accessed 1 June 2016].

3 CDC, Quarantine and Isolation, Available at http://www.cdc.gov/quarantine/ [Accessed 11 Jul 2017].

4 Lacey C. Abuse of quarantine authority, *Journal of Legal Medicine* 2003; 24 (2): 199‐214.

5 CDC. Notes on Interim US Guidance for Monitoring and Movement of Persons with Potential Ebola Virus Exposure 2014; Available at: http://www.cdc.gov/vhf/ebola/exposure/monitoring-and-movement-of-persons-with-exposure.html, [Accessed 5 Apr 2017].

6 Calain P, Poncin M. Reaching out to Ebola victims: Coercion, persuasion or an appeal for self‐sacrifice? *Social Science and Medicine* 2015; 147: 126‐133.

7 Ibid.

8 Lamond G. Coercion. In: LaFollette H, editor, *International Encyclopedia of Ethics* 2013. DOI: https://doi.org/10.1002/9781444367072.wbiee696

9 Feinberg J. *The Moral Limits of the Criminal Law*. New York: Oxford: Oxford University Press; 1989.

10 Buzan B, Waever O, de Wilde J. *Security: A New Framework for Analysis*. London: Lynne Rienner; 1998.

11 WHO 2003, Update 58 – *First Consultation on SARS epidemiology, travel recommendations for Hebei Province (China), situation in Singapore*, http://www.who.int/csr/sars/archive/2003_05_17/en/

12 Day T, et al. When is quarantine a useful control strategy for emerging infectious diseases? *American Journal of Epidemiology* 2006; 163 (5): 479‐85.

13 Bensimon C, Upshur R. Evidence and Effectiveness in Decisionmaking for Quarantine. *Am J Public Health* 2007; 97(Suppl 1): S44–S48; Bondy SJ, et al. Quantifying the impact of community quarantine on SARS transmission in Ontario: estimation of secondary case count difference and number needed to quarantine. *BMC Public Health* 2009; 9: 488; Barbera J, et al. Large‐scale quarantine following biological terrorism in the United States: scientific examination, logistic and legal limits, and possible consequences. *JAMA* 2001;286 (21):2711‐7; Schabas R. Severe acute respiratory syndrome: Did quarantine help? *Can J Infect Dis Med Microbiol* 2004; 15(4): 204.

14 UN Developing Program. *Human Development Report*:* New Dimensions of Human Security*. New York, Oxford University Press; 1994.

15 Elbe S. HIV/AIDS: The International Security dimension, In: Elke Krahmann, editor, *New Threats and New Actors in International Security*. New York: Palgrave; 2005.

16 Feldbaum H et al. Global health and national security: the need for critical engagement, *Medicine, conflict and survival* 2006; 22(3):192‐198.

17 Price‐Smith AT. *The health of nations: infectious disease, environmental change, and their effects on national security and developments*, Cambridge MA: MIT Press; 2002.

18 World Bank 2016. *World Bank Group Ebola Response Fact Sheet*, Available at http://www.worldbank.org/en/topic/health/brief/world-bank-group-ebola-fact-sheet, [Accessed 11 Jul 2017].

19 Lee JW, McKibbin W. Estimating the global economic costs of SARS, in Knobler, S. et al (eds.), *Learning from SARS: Preparing for the Next Disease Outbreak: Workshop Summary*, Washington, DC: National Academic Press; 2004, Available at http://www.ncbi.nlm.nih.gov/books/NBK92473/ [Accessed 11 Jul 2017].

20 Jackson F. Decision‐theoretic consequentialism and the nearest and dearest objection, *Ethics* 1991; 101 (3): 461‐482.

21 Gostin L. *Public Health Law: Power, Duty, Restraint*. Berkeley, CA: University of California Press; 2001.

22 Presidential Commission for the Study of Bioethical Issues 2015. *Ethics and Ebola. Public Health Planning and Response,* Available at https://bioethicsarchive.georgetown.edu/pcsbi/sites/default/files/Ethics-and-Ebola_PCSBI_508.pdf, [Accessed 11 Jul 2017]; Childress JF, et al. Public health ethics: mapping the terrain. *J Med Ethics* 2002; 30(2):170‐178.

23 Childress et al. *op. cit.,* note 22.

24 Tadros V. *The Ends of Harm. The Moral Foundations of Criminal Law*. New York: Oxford University Press; 2011.

25 Singer P. Famine, affluence, and morality. *Philosophy and Public Affairs* 1972; 1 (3): 229‐243.

26 Calain, Poncin, *op. cit*., note 6: 129.

27 Kutalek R, et al. Ebola interventions: listen to communities. *Lancet* 2015; 3:e131.

28 ACAPS 2016. *WASH in Guinea, Liberia, and Sierra Leone. The Impact of Ebola*, Available at https://www.acaps.org/special-report/wash-guinea-liberia-and-sierra-leone-impact-ebola, [Accessed 11 Jul 2017].

29 Singer, *op.cit*., note 25: p.230, emphasis added.

30 Scanlon T. *What We Owe to Each Other*, Cambridge, MA: Harvard University Press; 1998;224.

31 Savulescu J. Future people, involuntary medical treatment in pregnancy, and the duty of easy rescue. *Utilitas* 2007: 19(1): 1‐20: 10.

32 Upshur RE. Principles for the justification of public health intervention. *Canadian Journal of Public Health* 2002; 93:101‐103; Viens AM, Bensimon C, Upshur RA. Your liberty or your life: reciprocity in the use of restrictive measures in contexts of contagion. *Journal of Bioethical Inquiry* 2009;6: 207‐217.

33 Silva D, Smith M, Limiting rights and freedoms in the context of Ebola and other public health emergencies: how the principle of reciprocity can enrich the application of the Siracusa Principles, *Health and Human Rights Journal* 2015;1 (17): 52‐57; p. 53.

34 Ibid, p 54.

35 WHO 2015, *Stopping Ebola: it takes collaboration to care for a village*, Available at http://www.who.int/features/2015/stopping-ebola-in-kambia/en/, [Accessed 11 Jul 2017].

